# Supplementary material for: In Vivo Two-Photon Imaging Analysis of Dynamic Degradation of Hepatic Lipid Droplets in MS-275-Treated Mouse Liver
Source: Int J Mol Sci. 2022 Sep 1;23(17):9978. doi: 10.3390/ijms23179978 (PMC9456374; doi:10.3390/ijms23179978)
Supplement: Supplementary file 1 [file ijms-23-09978-s001.zip › Supplementary materials_revised.pdf]

In Vivo Two-Photon Imaging Analysis of Dynamic Degradation of Hepatic Lipid Droplets in MS-275 Treated Mouse Liver

Chang-Gun Lee†, Soo-Jin Lee†, Seokho Park†, Sung-E Choi, Min-Woo Song, Hyo Won Lee, Hae Jin Kim, Yup Kang, Kwan Woo Lee, Hwan Myung Kim, Jong-Young Kwak*, In-Jeong Lee* and Ja Young Jeon*

Supplementary Video S1. Live imaging of lipid droplets (LDs) in obese mouse liver tissue.

Supplementary Video S2. Live imaging of lipid droplets (LDs) and lysosomes in obese mouse liver tissue.

Supplementary Video S3. Live imaging of lipid droplets (LDs) and lysosomes in obese mouse liver tissues treated with DMSO or MS-275.
