# Supplementary material for: In Vivo Two-Photon Imaging Analysis of Dynamic Degradation of Hepatic Lipid Droplets in MS-275-Treated Mouse Liver
Source: Int J Mol Sci. 2022 Sep 1;23(17):9978. doi: 10.3390/ijms23179978 (PMC9456374; doi:10.3390/ijms23179978)
Supplement: Supplementary file 1 [file ijms-23-09978-s001.zip › Supplementary Table_S1.pdf]

Supplementary Table S1. Primer sequences used in this study

Name	Forward & Reverse (5' to 3')	Supplier
LIPC	F-TGCGCTGAAGTCCCGACAGT<br>R-CCACGTCCTGGCCCACAATA	Bioneer, Korea
PNPLA2	F-TCAGGCGAGAGTGACATCTG<br>R-CGAAGGTTGAACTGGATGCT	Bioneer, Korea
LIPE	F-TTGCAGGGGACAGTGCAGGT<br>R-GATGCCATCTGGCACCCTCA	Bioneer, Korea
MGLL	F-ACCAGCATGTCCAGCCCCTT<br>R-ACTGGAAGCCCAGTGGCACA	Bioneer, Korea
APRT	F-GCCTCTTGGCCAGTCACCTGA<br>R-CCAGGCTCACACACTCCACCA	Bioneer, Korea
TFAM	F-GCACTTGAAATGTGGGGAGT<br>R-CCTAACCTACACCCCTGCAA	Bioneer, Korea
NRF1	F-AGGTGGTGACCTTGGAACAG<br>R-GGCTTTTTGGGACAGTGAAA	Bioneer, Korea
NRF2	F-TTCGTTAAAGGGGAGGGACT<br>R-GGAAAGGCACAGAGAGCATC	Bioneer, Korea
PGC1α	F-GTCATTCGGGAGCTGGATGG<br>R-CAACCAGAGCAGCACACTCT	Bioneer, Korea
PPARα	F-AGAAGTTGCAGGAGGGGATT<br>R-TTGAAGGAGCTTTGGGAAGA	Bioneer, Korea
MCAD	F-GATCGCAATGGGTGCTTTTGATAGAA<br>R-AGCTGATTGGCAATGTCTCCAGCAAA	Bioneer, Korea
CPT1b	F-CCCCAGGCTGCAGAAATACC<br>R-TTTGCCTGGGATGCGTGTAG	Bioneer, Korea
UCP1	F-ACTGCCACACCTCCAGTCATT<br>R-CTTTGCCTCACTCAGGATTGG	Bioneer, Korea
FASN	F-CCTGGATAGCATTCCGAACCT<br>R-AGCACATCTCGAAGGCTACAC	Bioneer, Korea
SCD1	F-CTCCAGTTCTTACACGACCA<br>R-AGCCACGGCGGGAATTGTGA	Bioneer, Korea
ACACA	F-ATCGACACTGGCTGGCTGGA<br>R-GCCCCACACACAACTCCCAA	Bioneer, Korea
DGAT	F-TGGTGTGTGGTGATGCTGATC<br>R-GCCAGGCGCTTCTCAA	Bioneer, Korea
SREBP1c	F-GCTGTTGGCATCCTGCTATC<br>R-ATGCTGGAAGTGACGGTGGT	Bioneer, Korea
FGF21	F-ACCCCAGGTTACATCATCCA<br>R-GCGCCTGAACCATGATATTT	Bioneer, Korea
